# Floodplain inundation and lateral connectivity promote productivity in a managed river ecosystem

**DOI:** 10.1002/eap.70146

**Published:** 2025-11-23

**Authors:** Shruti Khanna, Catarina Pien, Pascale Goertler, Lauren Yamane, Elizabeth Stumpner, Jereme William Gaeta, Dylan Chapple, Mattea Berglund, Ryan Peek

**Affiliations:** ^1^ Interagency Ecological Program California Department of Fish and Wildlife Stockton California USA; ^2^ California Department of Water Resources Sacramento California USA; ^3^ United States Bureau of Reclamation Sacramento California USA; ^4^ Delta Stewardship Council Sacramento California USA; ^5^ United States Fish and Wildlife Service Lodi California USA; ^6^ California Department of Fish and Wildlife, Water Branch West Sacramento California USA; ^7^ Center for Watershed Sciences, University of California Davis California USA

**Keywords:** chlorophyll, connectivity, estuary, floodplain, flow, modeling, temperature

## Abstract

River‐floodplain ecosystems near urban centers are heavily engineered for flood protection and water delivery, which has led to a loss of lateral hydrologic connectivity between rivers and their floodplains. This study has two objectives: (1) Does increased lateral connectivity resulting from floodplain inundation increase chlorophyll *a* biomass? (2) Does that bump in chlorophyll *a* get transported downstream? The San Francisco Estuary in California, USA, has a robust and long‐term monitoring network for water quality. We integrated water temperature, chlorophyll *a*, flow, and floodplain inundation data from multiple sources creating a continuous dataset with fine temporal resolution spanning two decades. We used a consistent generalized additive mixed model structure across three regions: the floodplain, the mainstem of the river adjacent to the floodplain, and the section of the river downstream from both the floodplain and mainstem. We found that when the floodplain is not inundated, chlorophyll *a* biomass is mainly influenced by water temperature. However, when the floodplain is laterally connected during periods of inundation, water spreads over a larger surface area in the floodplain, flows decrease and water temperatures increase creating favorable conditions for chlorophyll *a* production. High flows during the flood pulse quickly transport chlorophyll *a* downstream, flushing the estuary with food. Under optimal conditions, tidal mixing in the downstream portion of the estuary can continue to boost chlorophyll *a* biomass in the system even after the flood waters have retreated. This study can guide the design, enhancement, and management of water conveyance structures to meet environmental flow regulations and to benefit the estuarine food web.

## INTRODUCTION

Hydrologic connectivity in river‐floodplain ecosystems has been altered by management of watersheds worldwide with far‐reaching implications for ecosystem health and services (Tockner et al., 2010). Lateral hydrologic connectivity is the movement of water, organic and inorganic matter, nutrients, and organisms between rivers and adjacent floodplains (Aftabuddin et al., 2017; Keeley et al., 2022; Pringle, 2001), whereas longitudinal hydrologic connectivity is the upstream–downstream movement of water and material within the river channel (Covino, 2017; Keeley et al., 2022). Reductions in connectivity can impact sediment transport (Guida et al., 2015), nutrient dynamics (Kufel & Lesniczuk, 2014; Li et al., 2018, 2022), primary productivity (Kufel & Lesniczuk, 2014; Li et al., 2022; Mazur et al., 2021), secondary productivity (Casanova et al., 2009; Cloern, 2007; Valentine‐Rose et al., 2007), and biotic diversity (Bayen et al., 2021; Mazur et al., 2021; Zilli & Paggi, 2013), adversely affecting the health of aquatic systems worldwide.

Human engineering and water management practices have diminished the duration, timing, and extent of lateral connectivity events within river‐floodplain ecosystems. Under historical conditions, the land‐water interface was governed by the interaction between the timing and magnitude of precipitation and hydro‐geomorphic features on the landscape that determined patterns of flooding and residence time. Landscape manipulations for water supply and flood control, such as channelization, water diversions, and reclamation for agriculture, have reduced pulse flows, homogenized mainstem channels, and disconnected floodplains (Tockner et al., 2010). Water is often stored in upper watershed dams and released to meet human needs, altering natural flow patterns (Brown & Bauer, 2010; Kimmerer, 2004; Yarnell et al., 2015). Below dams, rivers are often controlled by levees and other structures to prevent flooding in human‐dominated landscapes, preventing lateral connectivity with floodplains and riparian areas. Loss of floodplain connectivity can reduce the residence time of water on floodplains leading to substantially reduced primary and secondary productivity (Benke, 2001; Cloern, 2007; Lehman et al., 2008).

The Flood Pulse Concept (FPC) introduced by Junk et al. (1989) identifies episodic periods of connectivity that create optimal conditions for productivity following floodplain inundation. The relevance of river‐floodplain connectivity to primary production in space and time has also been identified in the Riverine Ecosystem Synthesis proposed by Thorp et al. (2006) and the River Wave Concept (Humphries et al., 2014). Many of the ecological outcomes associated with lateral connectivity in floodplains result from changes in the speed at which water moves across the landscape, which influences water residence time. Slower flow across shallow environments results in longer transport and residence times, and supports biological processes like primary production and carbon transport (Lucas & Thompson, 2012; Tockner et al., 1999). Alternatively, high flows during floods can decrease water transport time reducing biomass accumulation and transporting material quickly downstream (Reynolds & Descy, 1996). Optimal flow conditions can lead to both local increases in biomass (Howarth et al., 2000; Sommer et al., 2004), and export of productivity to adjacent areas (Cloern, 2007).

Flow conditions may increase primary productivity by optimizing light, water temperature, and nutrients during and following periods of lateral connectivity (Amoros & Bornette, 2002). In shallow environments, light availability often increases with decreasing flows and favors phytoplankton growth (Roach et al., 2014). Water temperature is a key variable for productivity and is moderated by the air and water interface in deeper channels, and by the air and land interface in shallow environments (Enright et al., 2013). Water temperature also affects phytoplankton community composition and grazing rates in managed estuarine systems (Cloern, 2017; Lehman, 2022). The presence of nutrients—together with the abundance of grazers—is known to control phytoplankton biomass and taxonomy in river corridors and estuaries (Glibert et al., 2016; Kraus et al., 2019; Lucas et al., 2016). Given that primary productivity is the foundation of the pelagic food web, lateral connectivity that supports autochthonous organic matter production and transports material downstream will further support higher trophic levels in downstream pelagic habitats (Cloern, 2007; Valentine‐Rose et al., 2007).

Techniques for reconnecting floodplains to their rivers for flood control and other uses date back to at least the eighteenth century (Teramura & Shimatani, 2021). Early efforts primarily aimed to protect people and infrastructure from flooding (Serra‐Llobet et al., 2021). As the ecological advantages and services of floodplains became better understood, flood control infrastructure in heavily modified hydrologic systems increasingly emphasized enhancing lateral connectivity between rivers and floodplains, supporting the restoration of food webs and other ecosystem services (Ahearn et al., 2006; Jeffres et al., 2020; Serra‐Llobet et al., 2021, 2022; Teramura & Shimatani, 2021). Examples of such projects span globally, from open levee backwater systems on Japan's Matsuura River and the Mississippi River system in the United States (Serra‐Llobet et al., 2021; Teramura & Shimatani, 2021), to levee setback projects on the Elbe and Isar Rivers in Germany and the Bear and Feather Rivers in California, United States (Serra‐Llobet et al., 2022), and to the flood bypasses on the Mississippi and the Sacramento River systems in the United States, and the Yangtze River in China. These projects represent a continuum of flood control, ecological restoration, and recreational benefits (Serra‐Llobet et al., 2021). Projects built with the objective of offering multiple benefits to society require more investment in land and likely more negotiation with stakeholders in the region. Hence, it is important to be able to quantify the advantages offered by these large projects as opposed to building single‐purpose projects, such as concrete bypass tunnels that may offer flood protection for much less investment (Serra‐Llobet et al., 2021).

The objective of this study is to explore how an engineered flood bypass influences chlorophyll *a* biomass and its transport downstream during periods of flood inundation. To date, most studies examining the relationship between lateral connectivity and primary and secondary production in the river ecosystem have been conducted over a single year comparing wet and dry seasons (Casanova et al., 2009; Jeffres et al., 2020; Lehman et al., 2008; Zilli & Paggi, 2013). While some studies use longer time series of discharge or water levels, the corresponding productivity is still measured over a single sample year (Li et al., 2018, 2022) or a few years (Mazur et al., 2021).

The San Francisco Estuary (SFE) in California, USA, is a tidal estuary and river delta with an extensive monitoring network producing data over the past six decades which includes continuous monitoring stations of river discharge and water quality (cdec.water.ca.gov; https://waterdata.usgs.gov/ca/nwis/sw), and discrete boat surveys of water quality, zooplankton, and fish (iep.ca.gov/Data/IEP-Survey-Data). The SFE also includes an engineered flood bypass within the historical floodplain of the Sacramento River called the Yolo Bypass. This synthesis effort brings together water temperature, flow, inundation, and chlorophyll *a* biomass data from 1999 to 2019 from the Yolo Bypass floodplain region, the mainstem of the Sacramento River running parallel to the floodplain, and the downstream region where both influences mix, integrating, for the first time, a continuous dataset with fine temporal resolution spanning two decades (Pien et al., 2023).

This unique integrated dataset allows us to examine how lateral connectivity affects chlorophyll *a* (as an indicator of primary productivity) and its transport downstream, mediated by water temperature and flow. Specifically, we asked the following questions:

1. How do flow and water temperature influence chlorophyll *a* biomass when the floodplain is dry? Do these relationships differ among regions?
2. Does the floodplain exhibit higher productivity compared with the mainstem river when the floodplain is active and lateral connectivity is restored? Does duration of inundation influence productivity?
3. Can increased lateral connectivity lead to improved longitudinal connectivity through transport of chlorophyll *a* downstream of the floodplain?

## DATA AND METHODS

### Study site

**FIGURE 1:**
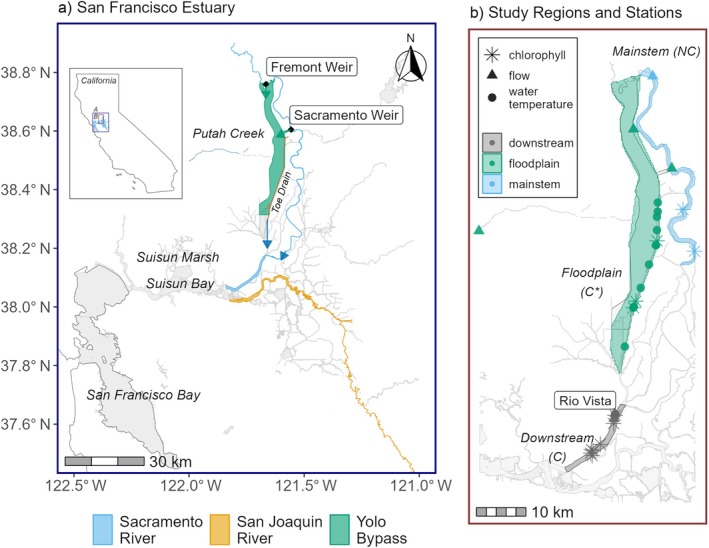
Study site. (a) The Sacramento River, San Joaquin River, and Suisun Marsh are highlighted as part of the San Francisco Estuary. Green arrows indicate lateral connectivity (from mainstem to floodplain) and blue arrows indicate longitudinal connectivity (from floodplain and mainstem to downstream). (b) Regions and stations used in analysis. C indicates connected, NC indicates not connected, and C* indicates conditionally connected (when inundation occurs). The flow stations outside of the floodplain region (green triangles) are used to calculate daily flow for the Yolo Bypass to account for water inputs from Putah Creek, Sacramento Weir and the Fremont Weir.

The SFE is located where two major river systems of California—the Sacramento and the San Joaquin—meet ocean tides traveling through the Golden Gate north of San Francisco (Figure 1a). The river systems meet in a network of leveed channels forming an inverted delta before flowing into the brackish waters of Suisun Bay. This region is called the Upper SFE. Nearly all rivers in the Upper SFE and its watershed, which drains 40% of California's freshwaters, have been channelized, and dams regulate flows (35%–65% of which is diverted for agricultural and urban use) (Kimmerer, 2004), and over 97% of wetlands in the system have been destroyed since the 1800s (Robinson et al., 2014). These changes led to greatly reduced primary productivity compared with historic conditions (Cloern et al., 2021) and the decline of a number of fish species, including the endangered winter run Chinook salmon (*Oncorhynchus tshawytscha*) and Delta smelt (*Hypomesus transpacificus*) (Moyle et al., 2016; Yoshiyama et al., 1998), which are currently the focus of large‐scale wetland and floodplain restoration and management actions.

The Yolo Bypass, located in the northeast Delta, is a 24,000‐ha partially leveed floodplain basin, fed by the Sacramento River via the Sacramento Weir (completed in 1916), the Fremont Weir (completed in 1929), and additional western tributaries, most notably Putah Creek (Figure 1a). It is designed to be inundated with freshwater during periods of high watershed outflow and drain through the Toe Drain, an engineered perennial channel along its eastern edge (Grimm & Lund, 2016). During wet periods with high flows in the mainstem Sacramento River, water overflows at the two weirs and enters the bypass. However, during dry periods without flows, aquatic habitat in the Yolo Bypass is reduced to the Toe Drain (Figure 1a). Thus, there is no continuous lateral connection between the mainstem and the flood bypass. Freshwater flows in parallel through the mainstem and the bypass, merging below the southern extent of the floodplain. Freshwater and tidal currents further mix and disperse materials, forming variable exchange and residence time zones that can structure phytoplankton communities and create “hydrodynamic habitats” (Smits et al., 2023; Stumpner et al., 2020; Young et al., 2021). Furthermore, State and Federal water project exports and agricultural diversions also affect flow in the system (Hartman et al., 2024). The study area experiences a Mediterranean climate characterized by a high degree of interannual variability and a range of flow conditions across years (Dettinger et al., 2016), thus, long‐term data are essential for capturing the range of conditions in the system (Hartman et al., 2024).

To determine whether floodplain lateral connectivity affects chlorophyll *a* biomass, we compared productivity across three different regions: (1) the mainstem of the Sacramento River adjacent to the floodplain (hereafter mainstem), (2) the Yolo Bypass floodplain (hereafter floodplain), and (3) the Sacramento River downstream of the confluence of the floodplain and the mainstem (hereafter downstream, Figure 1b).

### Data integration

Multiple datasets from different sources were compiled and integrated for this study (Pien et al., 2023). For detailed source information and description of the datasets, see Appendix S1. Chlorophyll *a* data (Appendix S1: Table S2) were used as the response variable and water temperature, shortwave radiation, flow, and floodplain inundation were explored as potential covariates (Appendix S1: Table S3). We explored 1‐week moving average windows of water temperature and shortwave radiation with the last day of the window corresponding to the date of the chlorophyll *a* measurement. Three floodplain inundation metrics were explored: inundation duration (number of days inundated), inundation factor (a categorical variable with the following levels: none, short: ≤21 days inundation, and long: >21 days inundation), and inundation (a binary variable with a value of 1 when inundated and 0 for non‐inundated). For a detailed description of inundation metrics for all years included in the study, see Appendix S1: Figure S1, Table S3. We tested all covariates for normality and collinearity (Appendix S1: Figure S2). Continuous variables such as daily flow and chlorophyll *a* were transformed using natural log to maximize normality (Appendix S1: Figure S3). Collinearity was evaluated via variation inflation factor (threshold value <3), following the methods reported in Zuur et al. (2010).

The time period analyzed was filtered to periods during which floodplain inundation had historically occurred in the Yolo Bypass (December to May). Covariates were combined into a single data frame. Approximately 2% of the flow values were missing and were replaced using a linear regression with an upstream station (USGS‐11447905, Sacramento River below Georgiana Slough). Any daily data gaps that could not be filled by the model were imputed with the imputeTS R package (Moritz & Bartz‐Beielstein, 2017). chlorophyll *a* data were then joined with daily covariates by date and assigned to the three regions, so each chlorophyll *a* value had a matching regional covariate value for flow, inundation, shortwave radiation, and water temperature. If there were multiple values for a single date and station for chlorophyll *a* (<1%), we assigned the mean of those values to that date. Days with no chlorophyll *a* data were excluded from the integrated dataset. This resulted in the dataset spanning March 9, 1999 to December 27, 2019 (Pien et al., 2023). This integrated dataset is visualized in Appendix S1: Figure S4.

### Study design and model selection

We hypothesized that increased lateral connectivity due to floodplain inundation would boost production of chlorophyll *a* within the floodplain. We expected an increase in chlorophyll *a* in the floodplain region (conditionally connected) during inundation periods, no corresponding increase in the mainstem region (not connected), and a muted increase in the downstream region (connected) due to partial transport of productivity from both floodplain and mainstem regions. Hence, we designed our study as a comparison of the same model fitted to the three different regions. To determine the most optimal model structure and the specific covariate used to represent temperature and inundation, we fitted our model to the floodplain region, where lateral connectivity likely has the strongest effect, and used forward selection to identify optimal model structure. We included covariates based on a conservative threshold of a reduction in the Bayesian Information Criteria of at least 7 (Burnham & Anderson, 2004). Following this method, we selected water temperature as the temperature metric and inundation factor as the floodplain inundation metric. We confirmed that the variance inflation factor was less than three for the covariates included in the final model. We also confirmed that the residuals did not show temporal autocorrelation and, therefore, we did not need to account for autocorrelation in the variance structure. After identifying the optimal model structure in the floodplain region, we applied the same model structure to the mainstem and downstream regions. We used this framework to explicitly compare model results among regions to test whether (1) the floodplain region exhibited higher productivity (as measured by chlorophyll *a*) than the mainstem region and, if evident, (2) whether the increased productivity signal continued into the downstream region (Figure 1).

Preliminary exploration with generalized linear models identified non‐linear relationships between the covariates and chlorophyll *a* that were not accounted for in traditional linear modeling frameworks. Hence, we explored the non‐linear generalized additive mixed model (GAMM) structure leveraging tensor products. The final models for the three regions were fit with a tensor product of flow and water temperature by inundation factor (equation 1). Tensor products allow for interactions between non‐linear covariates (flow and water temperature), through the application of a separate marginal smoothness penalty per covariate (Wood, 2017). The potential effect of sampling station was accounted for by including the random effect of sampling station (Figure 1b). The grand‐mean model predictions can, therefore, be interpreted as the regional chlorophyll *a* after accounting for sampling station. Each regional model followed the following structure given in equation 1:

(1) $$\text{Chlorophyll}\;a˜\mathrm{te}\left(Q,\mathrm{WT},\mathrm{by}=I\right)+I+s\left(S,\mathrm{bs}=\mathrm{re}\right)$$

where te = tensor product, re = random effect, chlorophyll *a* = log_e_(chlorophyll *a*) (in micrograms per liter), *Q* = log_e_(daily mean flow [cfs]), WT = 7‐day mean of daily mean water temperature (in degree Celsius), *S* = station, and *I* = inundation factor with three values (none: no inundation, short: ≤21 days of inundation to date of observation, and long: >21 days of inundation to date of observation).

We validated regional models via assessment of residuals and covariates, residuals, and fitted values and examining temporal autocorrelation in residuals and residual histograms (Gelman & Hill, 2006). Given the complex nature of non‐linear models, particularly those developed with tensor products, we took a simulation approach to evaluate our hypotheses. Specifically, we simulated datasets of our covariates restricting them from 5 to 95 percentiles of existing values in our data within each region and predicted chlorophyll *a* for the simulated data matrices using the “tidymv” package (version 3.4.2).

Statistical analyses were performed using R Statistical software (R Core Team, 2023; version 4.2.2) following procedures described in Gelman and Hill (2006), Pedersen et al. (2019), and Zuur et al. (2009). The df (*k* factor) for all smoothers, random effects, and tensor products were adequate based on the *k*‐index value (Wood, 2017) as reported by the gam.check function from the “mgcv” package (version 1.8.39). We estimated 95% CIs of grand mean model predictions through the “emmeans” package (version 1.10.1). The prediction matrix was used to generate approximate pointwise confidence intervals for comparisons among smooths following methods described by Rose et al. (2012).

## RESULTS

### Data summary statistics

**FIGURE 2:**
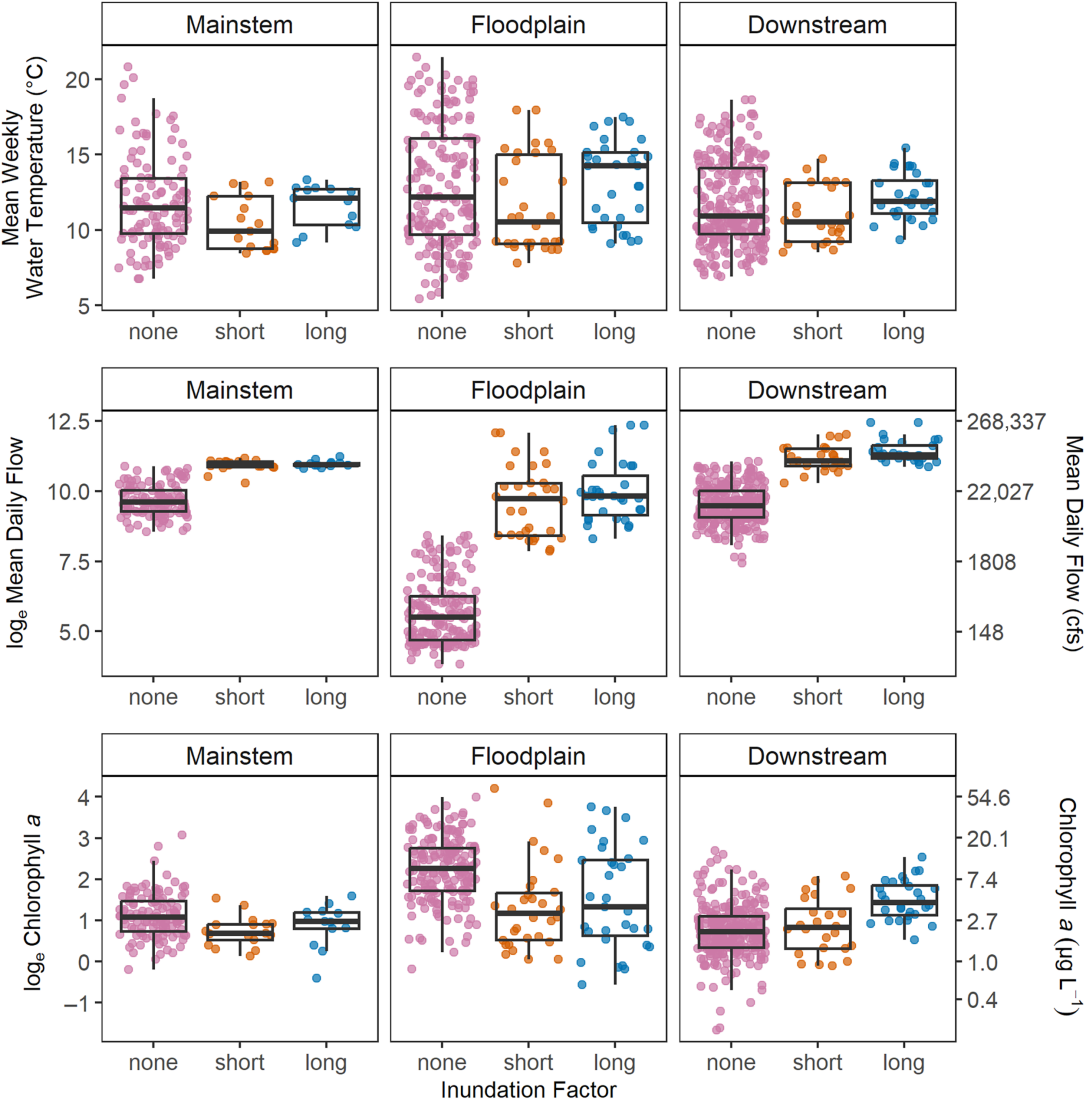
Boxplots of raw data used in the model. Data are organized by region and inundation factor.

During the study period, water temperature values were more variable during non‐inundated periods compared with inundated periods across all three regions, but the range of values largely overlapped (Figure 2; Table 1). Mean flows were lowest during non‐inundated periods and significantly higher across all regions when the floodplain was inundated (Table 2). In fact, flow ranges during non‐inundation periods barely overlapped with the range of values during inundated periods, making it difficult to disambiguate the effect of flow versus inundation (Figure 2; Table 1). The lowest flow values occurred in the floodplain during non‐inundated periods (when only the Toe Drain has flowing water), and the highest flow values occurred in the downstream region which receives inflow from both the floodplain and the mainstem (Table 1). The highest values and the widest range of chlorophyll *a* values occurred in the floodplain regardless of inundation status (Table 1). The highest variability of covariate values occurred during the non‐inundated time period because this period also includes dry years that are not represented in inundated periods. During inundation, the floodplain experienced a large amount of flow variability compared with other regions (Figure 2).

**TABLE 1:** Means and 5–95 percentiles of mean weekly water temperature (WT), daily flow, and chlorophyll *a* (Chl *a*) for all three regions and inundation factors and the number of observations for each.

Covariate	Mainstem region			Floodplain region			Downstream region		
Inundation	None	Short	Long	None	Short	Long	None	Short	Long
WT (°C), 5th percentile	7.7	8.6	9.4	7.3	8.7	9.3	7.8	8.7	10.2
WT (°C), mean	11.9	10.4	11.6	12.9	11.6	13.2	11.8	11.0	12.2
WT (°C), 95th percentile	17.4	13.1	13.0	19.6	17.0	17.2	16.9	13.9	14.3
Flow (cfs), 5th percentile	6756	35,100	50,000	86	3209	6204	4570	37,330	58,625
Flow (cfs), mean	18,358	55,535	58,062	635	32,444	41,519	17,060	77,867	99,754
Flow (cfs), 95th percentile	42,630	66,660	70,620	2867	135,474	210,594	47,420	155,800	221,600
Chl *a* (μg L^−1^), 5th percentile	1.4	1.3	1.0	2.4	1.1	0.8	0.9	0.9	2.4
Chl *a* (μg L^−1^), mean	3.6	2.2	2.7	12.3	7.8	9.5	2.6	3.0	
Chl *a* (μg L^−1^), 95th percentile	6.3	4.1	4.4	30.0	34.0	36.0	6.8	6.9	9.1
Number of observations (*N*)	114	17	13	176	30	31	257	24	28

**TABLE 2:** Pairwise comparisons for flow during different inundation periods (none, short, and long).

Region	Dunn's test (Dinno, 2017)	*Z*	*p*‐value (holm correction)
Mainstem	None‐short	−6.407	<0.001*
	None‐long	5.880	<0.001*
	Short‐long	0.151	0.440
Floodplain	None‐short	−8.441	<0.001*
	None‐long	9.045	<0.001*
	Short‐long	0.369	0.356
Downstream	None‐short	−7.571	<0.001*
	None‐long	8.864	<0.001*
	Short‐long	0.532	0.297

*Note*: Asterisk values indicate significant differences.

### Model results

**FIGURE 3:**
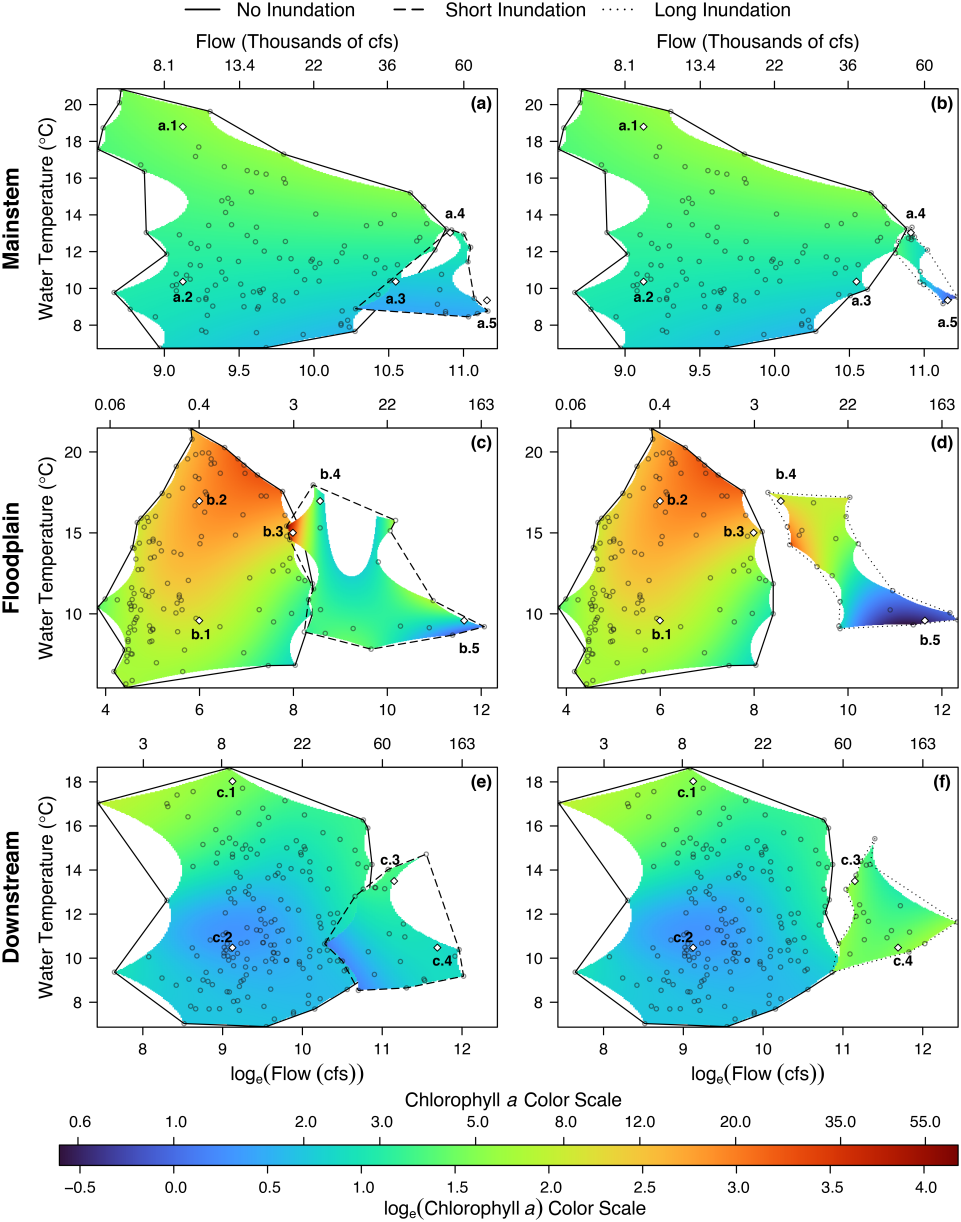
Simulated chlorophyll *a* values in micrograms per liter for flow and water temperature (a) in mainstem for none and short inundation, (b) none and long inundation, (c) in floodplain for none and short inundation, (d) none and long inundation, (e) in downstream for none and short inundation, (f) and none and long inundation durations. Data points from the input data are plotted as hollow points in flow‐temperature 2D covariate space. Model predictions are constrained to an alpha‐shaped convex hull encompassing the data cloud to preclude extrapolation.

Chlorophyll *a*, in general, was higher in the floodplain than in the other two regions (Table 1). However, in each of the three regions, chlorophyll *a* was influenced slightly differently by flow and water temperature mediated by inundation. For all three regions, when the floodplain was not inundated, chlorophyll *a* concentrations were positively correlated with water temperature and barely influenced by flow (Figure 3; solid line polygons).

**FIGURE 4:**
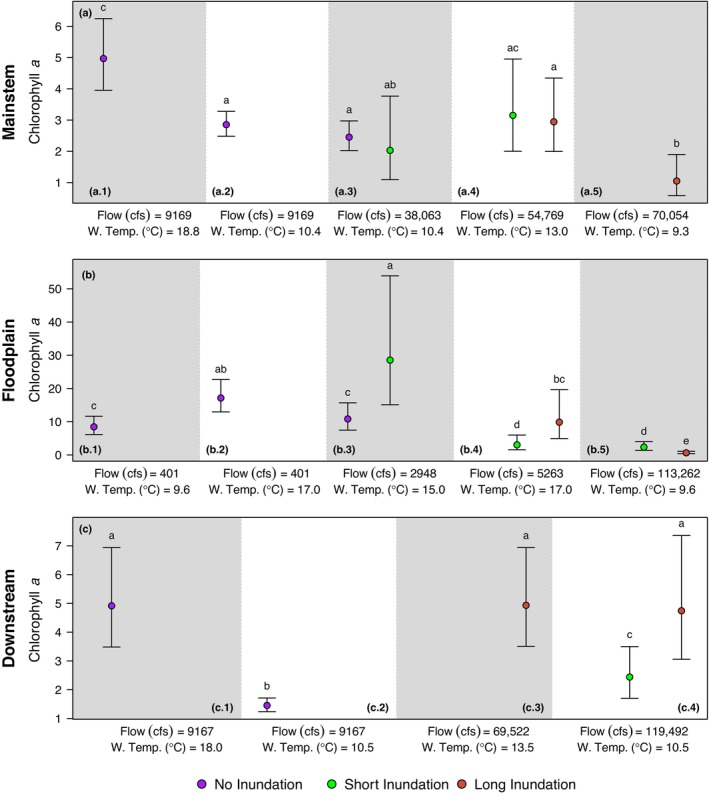
Point and uncertainty estimates for combinations of different flows, water temperatures, and inundation factor as simulated by (a) the mainstem model, (b) the floodplain model, and (c) the downstream model. Point estimates with different letters represent estimates significantly different from each other within each row of panels.

**FIGURE 5:**
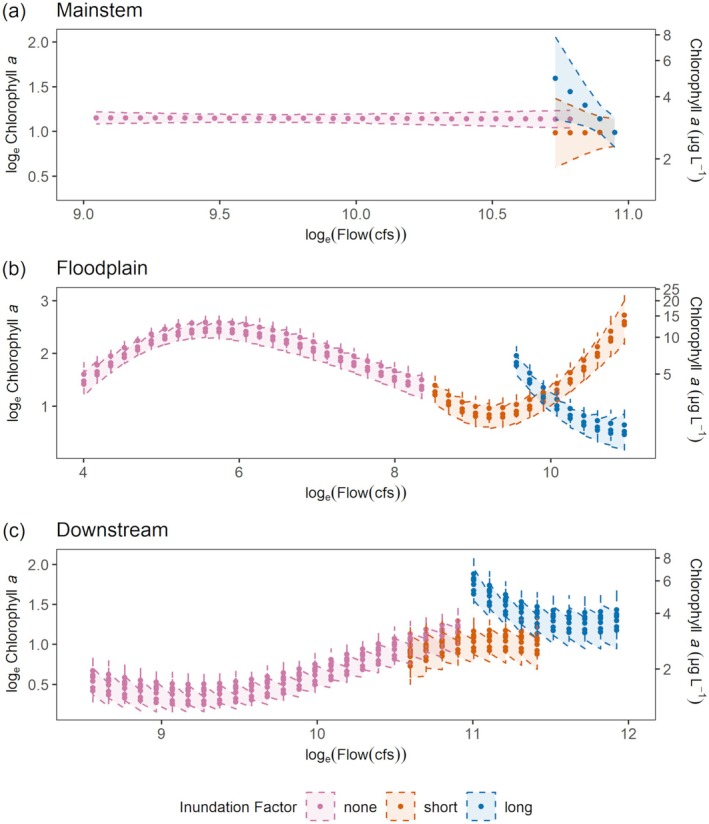
Predicted log_e_(chlorophyll *a*) at 12°C and the possible range of log_e_(flow) values in the dataset, for each region, (a) mainstem, (b) floodplain and (c) downstream, for none, short and long inundation. Multiple points for the same flow value indicate predicted values across multiple stations. Scales are not equivalent across the three panels.

Of the three regions, the mainstem model explained the least total deviance (33.4%; Table 3). During non‐inundated and short (≤21 days) duration inundation periods, the mainstem model indicates that water temperature is the main determinant of productivity (Figure 3a). At high water temperatures, chlorophyll *a* is higher and at low water temperatures, chlorophyll *a* is lower. For example, point estimates for chlorophyll *a* at ~9000 cfs when water temperature is 18.8°C are significantly higher than at 10.4°C (Figure 4a‐a.1,a.2). On the other hand, there is almost no change in chlorophyll *a* with flow (Figure 5a). For example, at a water temperature of 10.4°C, chlorophyll *a* is not significantly different as flow rises from ~9000 to ~38,000 cfs (Figure 4a‐a.2,a.3).

**TABLE 3:** *T* values for the three main models fitted to the three regions, deviance explained and adjusted *R* ^2^ by each regional model, and the number of observations available for each region and inundation type.

Region	Covariate	Estimate	SE	*T*	*p* value	Deviance explained	*R* ^2^ adjusted	*N*
Mainstem	Intercept	1.084	0.049	22.309	<0.001	33.4%	27.8%	114
	Short inundation	−0.171	1.668	−0.103	0.918			17
	Long inundation	2.707	2.450	1.105	0.271			13
Floodplain	Intercept	1.954	0.232	8.433	<0.001	65.4%	61.9%	176
	Short inundation	5.926	6.859	0.864	0.389			30
	Long inundation	15.020	6.328	2.374	0.019			31
Downstream	Intercept	0.847	0.067	12.575	<0.001	43.9%	39.2%	229
	Short inundation	−1.134	1.191	−0.952	0.342			24
	Long inundation	5.010	3.048	1.644	0.101			28

However, during long‐ (>21 days) duration inundation events, chlorophyll *a* is related to both flow and water temperature even in the mainstem, even though the mainstem is not connected to the floodplain. There is a sharp negative correlation between chlorophyll *a* and flow for the long‐duration inundation events as seen in Figure 5a. Furthermore, estimates for points a.4 and a.5 (Figure 4a) show that chlorophyll *a* is significantly higher in conditions with low flow and high water temperature compared with conditions with high flow and low water temperature.

The model for the floodplain region explained the most deviance compared with the other regions (65.4%; Table 3). In general, much higher values of chlorophyll *a* were observed in the floodplain compared with the mainstem and downstream regions (Figure 2; Table 1). During non‐inundation periods in the floodplain, chlorophyll *a* was mainly influenced by water temperature. Estimates of points b.1 and b.2 show that at the low flow of ~400 cfs, chlorophyll *a* was significantly higher at 17°C compared to 9.6°C (Figure 4b). However, the relationship was also mediated by flow (Figure 3c, solid polygon). For example, at a fixed water temperature of 12°C, chlorophyll *a* values first rise then fall as flow increases even during non‐inundated periods (Figure 5b).

During periods of long‐duration flooding, chlorophyll *a* was highest at high water temperatures and low flows and lowest at low water temperatures and high flows (Figure 3d). For example, chlorophyll *a* was significantly higher when flow was 5263 cfs and water temperature was 17°C compared to when flow was 113,262 cfs and water temperature was 9.6°C (Figure 4b‐b.4,b.5). This pattern is harder to observe in the short‐duration inundation period due to fewer data points in the center of the range of flow values, where the model fit is less reliable (Figure 3c, dashed polygon). Figure 4b also illustrates the effect of flood duration. At 15°C water temperature and ~3000 cfs, chlorophyll *a* was significantly higher during short inundation compared with non‐inundation periods (Figure 4b‐b.3). At 17°C and ~5000 cfs, chlorophyll *a* was significantly higher during long inundation compared with short inundation periods (Figure 4b‐b.4). However, at very high flows (>11,000 cfs), chlorophyll *a* decreased during long inundation flooding compared with short inundation flooding (Figure 4b‐b.5).

The downstream model explained 43.9% of total deviance in the data (Table 3). As with the other two regions, in non‐inundated periods, water temperature was the main determinant of chlorophyll *a*. For example, simulated point estimates for the same flow (~9000 cfs) but higher water temperature (18°C) were significantly higher than for a lower water temperature (10.5°C; Figure 4c‐c.1,c.2). The highest chlorophyll *a* values in the downstream region were observed during long inundation (Figure 2). During high flows (~120,000 cfs), a longer duration of inundation resulted in significantly higher chlorophyll *a* compared to a shorter duration of inundation (Figure 4c‐c.4).

## DISCUSSION

When the Yolo Bypass floodplain is inundated, water velocity decreases and residence time increases compared with the mainstem of the Sacramento River, stimulating primary and secondary production (Sommer et al., 2004). This heterogenous shallow aquatic habitat provides high‐quality rearing habitat for Chinook salmon and other native fish species (Feyrer et al., 2006; Henery et al., 2010; Sommer et al., 2007). To determine how lateral hydrologic connectivity affects chlorophyll *a* biomass in the floodplain and if this biomass is transported downstream, we explored the effect of flow and temperature on chlorophyll *a* contingent on inundation across all three regions using a consistent model structure.

### Effect of water temperature and flow

In the Upper SFE, higher flows generally co‐occur with lower temperatures (Bashevkin & Mahardja, 2022); however, we found that the relationship with chlorophyll *a* was more complex and spatially dependent. When the floodplain was not inundated (longitudinal connectivity alone), our results show that chlorophyll *a* concentrations were mainly driven by water temperature (Figure 3, solid polygons). Higher water temperatures can result in higher grazing rates of benthic invertebrates and zooplankton on phytoplankton (Cloern, 2017), however, we found that higher water temperature in all three regions resulted in higher observed chlorophyll *a*. It is possible that we did not observe a dampening in chlorophyll *a* concentrations due to high grazing rates because we constrained our study from December to May (historical inundation period), thus excluding peak summer temperatures. Flow had almost no impact on chlorophyll *a* in the mainstem and downstream regions but in the floodplain, higher‐than‐average flows strengthened the relationship between water temperature and chlorophyll *a* (Figure 3c, solid polygon). Thus, certain temperature and flow combinations can lead to higher chlorophyll *a* biomass, but the combination is unique to each region. For example, in the floodplain, where water velocity is considerably lower, higher chlorophyll *a* concentrations occur at lower temperatures relative to the other regions.

### Effect of inundation and region

In the mainstem, we expected inundation duration (long vs. short inundation) to make no difference in observed relationships between water temperature, flow and chlorophyll *a*. However, during long inundation periods (when the floodplain is inundated for more than 3 weeks), there is a negative relationship between flow and chlorophyll *a* in the mainstem (Figure 5a). This is likely because long‐duration floods only happen in years characterized by high flow across the watershed. Under high‐flow conditions, chlorophyll *a* produced in the mainstem is quickly advected downstream. This “washout” decreases residence time, preventing phytoplankton accumulation and has previously been observed in the estuary (Dugdale et al., 2012; Hammock et al., 2019; Lucas & Thompson, 2012). Moreover, high flows often occur with lower temperatures and higher sediment loads that limit conditions favorable for phytoplankton growth (Alpine & Cloern, 1988; Cole & Cloern, 1984, 1987).

In the floodplain, during periods of short‐ and long‐duration inundation, flow has a larger effect on chlorophyll *a* compared to non‐inundated periods (Figure 5b). However, the relationship between flow and chlorophyll *a* is different during short inundation periods compared to long inundation periods. During long‐duration floodplain inundation, as flow increased, chlorophyll *a* decreased (Figure 4b, red point estimates: b.4 and b.5). Meanwhile, for short inundation periods, as flow increased and water temperature decreased, there was no significant difference between point estimates of chlorophyll *a* (Figure 4b, corresponding green point estimates: b.4 and b.5), though results for the short‐duration flood inundation are less reliable for moderate flow values due to lack of data (Figure 3c, dashed polygon). Chlorophyll *a* was significantly lower at higher flows and may be attributed to the “washout” phenomenon where productivity is quickly transported downstream.

The downstream model shows mixed effects of patterns observed in the mainstem and in the floodplain as would be expected since water passes through both regions in parallel and converges downstream. Model fits for short and long inundation periods show that flow plays a role in addition to water temperature. Contrary to the floodplain and the mainstem region, chlorophyll *a* is higher with water temperature but also higher with flow (Figures 3e, dashed polygon and 5c). The highest chlorophyll *a* values were observed with moderate water temperatures and high flows in the downstream region. This is likely because elevated chlorophyll *a* at moderate water temperatures, is rapidly transported downstream from the mainstem and the floodplain regions during high flows. Subsequently, in the downstream region under unique conditions, tidal forcing may limit the advection of that productivity out of the estuary (Brown et al., 2024; Kimmerer, 2004). Once phytoplankton reach the downstream region, light and nutrient conditions can further favor algal growth.

**FIGURE 6:**
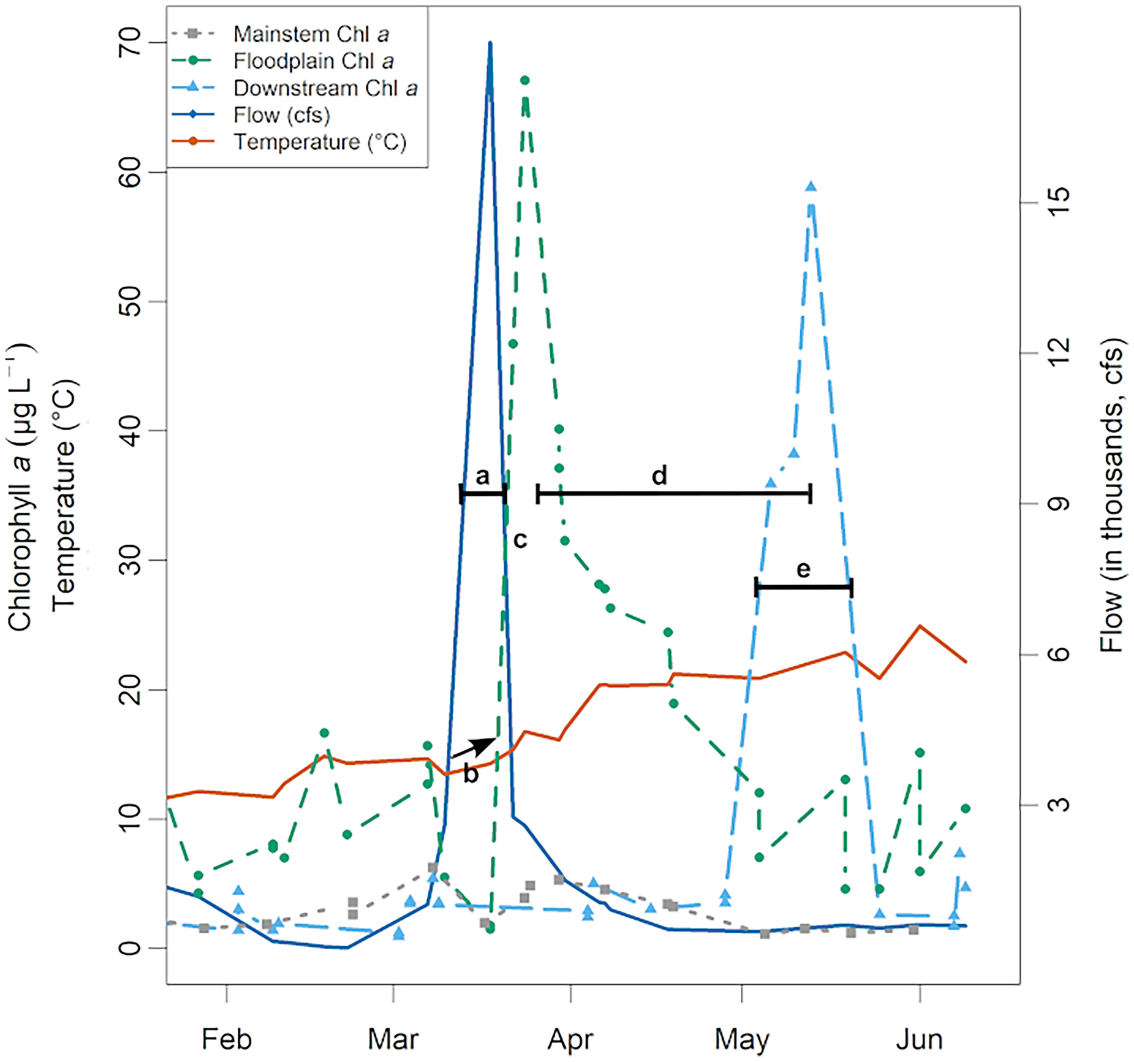
Example of a short inundation from 2016. (a) As the floodplain is inundated, (b) water temperature slowly rises as water sits on the floodplain baked by the sun. (c) This leads to a rise in chlorophyll *a* production in the floodplain. (d) As the flood recedes, the productivity is transported downstream increasing downstream production but depressing chlorophyll *a* levels in the floodplain. (e) Tidal mixing maintains higher chlorophyll *a* levels in the downstream region for some time.

**FIGURE 7:**
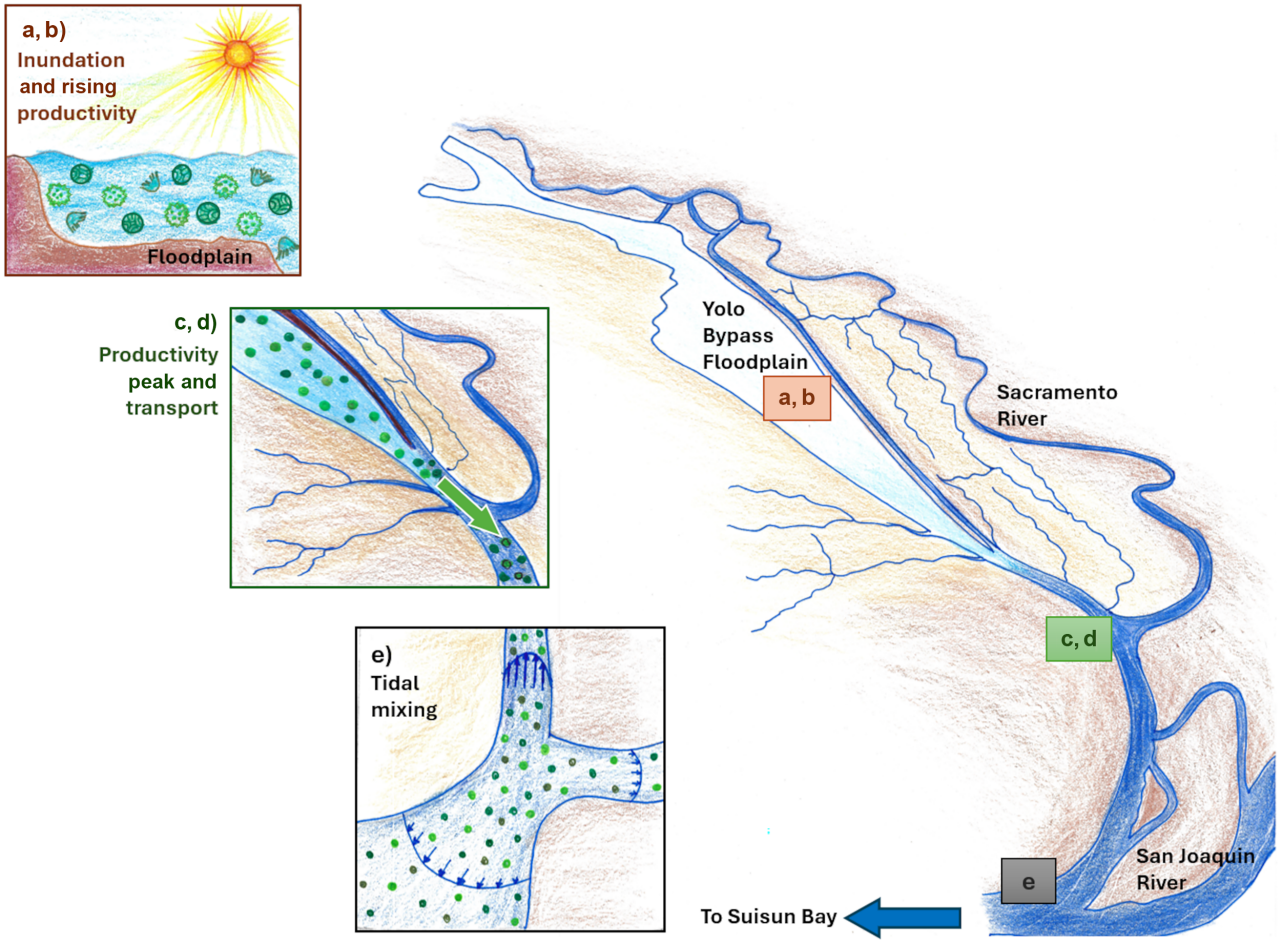
Conceptual framework of processes underlying observed patterns in Figure 6. (a, b) As the floodplain is inundated, the shallow depth of the water column is warmed by the sun and water temperature rises creating ideal conditions for a rise in chlorophyll *a* production. (c, d) During long inundation in wet years, high flows can immediately transport chlorophyll *a* downstream and during moderate flows, chlorophyll *a* is flushed downstream when the flood recedes. (e) Once downstream, tidal mixing and ideal nutrient conditions can potentially prolong the higher productivity levels. Figure illustration by Shruti Khanna.

During short inundation periods with moderate flows, the transport of chlorophyll *a* downstream is not immediately apparent. But it is likely that when the flood waters ebb, the chlorophyll *a* from the floodplain is flushed downstream providing the estuary a boost in productivity. The model structure we used in this study did not explore lags between the floodplain and downstream chlorophyll *a* biomass. But we looked closely at one example of a moderate flow short‐duration flood year, the 2016 water year (Figure 6) to follow the sequence of events from the start of the flood to the productivity boost downstream of the floodplain. We explain the chronology of events observed in 2016, and the relationships revealed by the GAMM models, through a conceptual model visualized in Figure 7.

### A conceptual framework

An inundated floodplain reduces water flow and greatly increases the area of shallow water exposed to sunlight within the river floodplain ecosystem (Figure 6a). This increases the water temperature on the floodplain relative to the mainstem (Figures 6b and 7). Light penetration in the shallow water column and higher water temperature together create ideal conditions for chlorophyll *a* production (Figure 6c). This bump in primary productivity likely travels up the food chain, increasing secondary and tertiary productivity (Ahearn et al., 2006; Jeffres et al., 2020). Depending on flow conditions, the floodplain chlorophyll *a* is either immediately transported downstream during high flows, or is flushed downstream later as flood waters recede (Figure 7).

The floodplain waters are diluted by the influx of mainstem water, but still show an increase in downstream biomass while depressing chlorophyll *a* levels in the floodplain. The year 2016 only had a short‐duration flood and moderate flows; hence the chlorophyll *a* from the floodplain was transported downstream only during the recessional limb of the hydrograph, as seen by the lag between peak chlorophyll *a* biomass in the floodplain and the mainstem (Figure 6d). Once this productivity pulse reaches downstream of the floodplain and mainstem, tidal influences get stronger. Tidal mixing, when concurrent with favorable water temperature and nutrient conditions, can sustain higher chlorophyll *a* levels in the downstream region for some time (Figure 6e).

## CONCLUSIONS AND FUTURE WORK

Given the demand for flood conveyance and freshwater flows for urban, agricultural, and environmental purposes, the manipulation of engineered systems is one option for creating and improving lateral connectivity. Typically, flood control infrastructure reduces the potential for lateral connectivity through dams, diversions, and levees; however, it can also be modified to increase lateral connectivity between the river and floodplain as a management strategy to improve the river‐floodplain ecosystem food web (Ahearn et al., 2006; Jeffres et al., 2020; Opperman et al., 2010). Such measures can increase primary productivity (Cloern et al., 2021), and help target endangered native species by providing both food web production and access to more complex rearing habitats and alternative migratory routes (Goertler et al., 2017; Sommer et al., 2020).

Our study explores how lateral connectivity, flow and water temperature mediate chlorophyll *a* biomass and exchange in three distinct regions of the Upper SFE using a synthesized dataset from the last two decades. The model results show that during dry periods, chlorophyll *a* is mainly influenced by water temperature, but during wet periods when lateral connectivity is high, flow and the duration of flooding also become important covariates and increase chlorophyll *a* within the floodplain region. Furthermore, it appears that this productivity boost travels downstream with high flows or when the flood ebbs and flushes the rest of the estuary with chlorophyll *a*. We propose a conceptual model for the sequence of events accompanying a flood pulse in a managed system like the upper SFE. A better understanding of how to effectively leverage such management solutions for multiple objectives (e.g., flood control and aquatic food supply) across variable conditions could be a powerful tool when applied to resource management in urbanized watersheds. Natural resource limitations require the balancing of many fish, wildlife, and water use objectives in adaptively managed landscapes. These model results describe the available data and relationships between temperature, flow and inundation at a broad scale, which could enhance the development of adaptive management plans for aquatic habitat restoration and water management for environmental benefits, such as efforts to optimize managed floodplains. For example, a recognition of the value of lateral connectivity and floodplain inundation has prompted the development of projects in the Yolo Bypass that would increase the inundated area during smaller flood pulses (Huntsman et al., 2024). A major engineering project that creates a notch in the Fremont Weir is poised to go into operation and will increase the frequency of flooding in Yolo Bypass during smaller high flow events in the upstream river systems (Grimm & Lund, 2016; Huntsman et al., 2024).

We identified four areas of future study to further develop understanding of the links between lateral conductivity in ecosystem productivity: nutrients, zooplankton dynamics, time lags, and other managed wetlands in the floodplain. Studies have shown that factors such as nutrient availability (Glibert et al., 2016; Parker et al., 2012; Strong et al., 2021) and the presence of invasive bivalves (Kraus et al., 2017; Lucas & Thompson, 2012) affect phytoplankton production. However, we were unable to explore the impact of nutrients because data available on nutrients were limited in length and frequency relative to the chlorophyll *a* dataset. Zooplankton grazing can also mask total chlorophyll *a* production because grazing rates increase in higher water temperatures (Jeffres et al., 2020). Taking zooplankton dynamics into consideration might provide a clearer picture of the relationship of primary production to lateral connectivity. Initially, we explored Bayesian methods of modeling chlorophyll *a* so that we could determine time lags between rising floods, chlorophyll *a*, and its transport downstream, but the Bayesian models did not explain as much of the variance as the GAMM models, and data were likely insufficient to determine the importance of different lags. However, lags undoubtedly play a role in the biological processes underlying floodplain primary production (Ahearn et al., 2006; Goertler et al., 2017; Kimmerer, 2004), and future studies should try to incorporate these into the modeling structure. Additionally, managed wetlands and rice fields in the Yolo Bypass and elsewhere have their own site‐specific dynamics but become connected to the Delta ecosystem during flood years, affecting nutrient conditions downstream. We did not consider the impact of these regions separately. Exploration of these research avenues will go a long way towards a holistic understanding of the effects of lateral connectivity on primary production in managed urban estuarine ecosystems.

## AUTHOR CONTRIBUTIONS

*Conceptualization*: All authors. *Data curation*: Catarina Pien, Pascale Goertler, Elizabeth Stumpner, Dylan Chapple, Mattea Berglund, and Ryan Peek. *Formal analysis, investigation, and validation*: Shruti Khanna, Catarina Pien, Pascale Goertler, Lauren Yamane, and Jereme William Gaeta. *Methodology*: Shruti Khanna, Catarina Pien, Pascale Goertler, Lauren Yamane, and Jereme William Gaeta. *Visualization*: Jereme William Gaeta, Pascale Goertler, and Catarina Pien. *Writing (original draft, review and editing)*: All authors. *Resources and program management*: Pascale Goertler and Dylan Chapple.

## CONFLICT OF INTEREST STATEMENT

The authors declare no conflicts of interest.

## Supporting information


Appendix S1.

## Data Availability

Data (Pien et al., 2023) are available in the Environmental Data Initiative Data Portal at https://doi.org/10.6073/pasta/ef0b914dbbb60d20466755dea95bf9bf. Code (Goertler et al., 2025) is available in Zenodo at https://doi.org/10.5281/zenodo.15485861.
