# Supplementary material for: Floodplain inundation and lateral connectivity promote productivity in a managed river ecosystem
Source: Ecol Appl. 2025 Nov 23;35(7):e70146. doi: 10.1002/eap.70146 (PMC12641158; doi:10.1002/eap.70146)
Supplement: Supplementary file 1 — Appendix S1. [file EAP-35-e70146-s001.pdf]

Journal: Ecological Applications

Appendix S1

Floodplain inundation and lateral connectivity promote productivity in the river ecosystem

Shruti Khanna, Catarina Pien, Pascale Goertler, Lauren Yamane, Elizabeth Stumpner, Jereme William Gaeta, Dylan Chapple, Mattea Berglund, Ryan Peek

Study Area

The San Francisco Estuary (SFE) is the largest estuary on the west coast of North and South America (https://www.epa.gov/sfbay-delta/about-watershed). The Sacramento River flowing in from the north, meets the San Joaquin River flowing in from the south in a network of leveed channels which form the SFE Delta (Figure 1). These rivers meet the tidal brackish water of the SFE, just east of Suisun Marsh (Figure 1a).

The Sacramento River is the larger of the two rivers, with a watershed of 70,000 km^2 (https://www.fisheries.noaa.gov/west-coast/about-us/sacramento-river-basin-california, USEPA, 2022) and average annual flows of 30,000 cfs (https://sacriver.org/explore-watersheds/sacramento-valley-subregion/), compared to 40,000 km^2 and 5000 cfs (https://www.waterboards.ca.gov/centralvalley/water_issues/swamp/sanjoaquin_river_basin/) for the San Joaquin River. The Sacramento River contains multiple bypasses, where river flows can be diverted during periods of high flow. The Yolo Bypass is a major bypass for the Sacramento River prior to meeting the estuary. In fact, both the river and the bypass drain into the SFE. The bypass is fed by the Sacramento Weir completed in 1916, and the Fremont Weir completed in 1929. The Yolo Bypass is operated for both flood control and ecosystem purposes, as the area mimics historical natural floodplain processes and has documented benefits for native fish species (Sommer et al. 2001a; b, 2005).

Data

To examine the impact of hydrologic connectivity on chlorophyll-a in context of other covariates, we integrated several datasets from multiple sources as described in detail in this section. To determine if chlorophyll-a concentrations increase within the floodplain during inundation, we compared primary production in the Yolo Bypass (floodplain region) to production in the main stem of the Sacramento River (mainstem region). To observe if this

increase in chlorophyll-a travels downstream, we included data from the section of the river downstream from both the river and the floodplain (downstream region).

Chlorophyll-a

Table S1. Chlorophyll-a data sources.

Region	Location	N	Dates	Sampling frequency	Source
Mainstem (Sacramento River)	Sacramento River at Sherwood Harbor (SHR) and Freeport (USGS-11447650)	144	2009/02/11 - 2019/12/27	Biweekly – monthly	CDWR, USGS CAWSC
Floodplain (Yolo Bypass)	Yolo Bypass Fish Monitoring Program Rotary Screw Trap, Lisbon Weir (LIS), and USGS-11455139	237	2009/02/11 - 2019/12/26	Biweekly – monthly	CDWR, USGS CAWSC
Downstream (Sacramento River)	657, 34, NZ068, 653, USGS-11455478, 16, D22	309	1999/03/09 - 2019/12/19	Biweekly - monthly	USGS CAWSC, USGS, CDWR, USBR

We used Chlorophyll-a as an indicator of phytoplankton abundance and biomass, our key response variable. Chlorophyll-a data were collected by multiple agencies (Table S1) and summarized into a daily dataset (one value per region per date, stations were selected randomly when multiple readings were available per date and region). Specifically, chlorophyll-a data were acquired from an integrated water-quality dataset (Bashevkin et al. 2023) and a dataset specific to the California Department of Water Resources Yolo Bypass Fish Monitoring Program (IEP et al. 2023). Chlorophyll-a data were restricted to the geographic extent of the model domain.

Flow data

River flow data in the mainstem and downstream regions were collected at the Sacramento River at Verona station (USGS 11445500) and Sacramento River at Rio Vista station (USGS 11455420), respectively. For both regions, daily mean values were retrieved from the National Water Information System (NWIS; U.S. Geological Survey, 2022) using the R package dataRetrieval (De Cicco et al. 2022). Because the downstream region is tidally influenced, daily mean flows retrieved from the NWIS database are calculated as net flow. Missing data in the downstream flow record were filled using a linear regression with an upstream station (USGS-11447905, Sacramento River below Georgianna Slough). The coefficient of determination

between the two stations was strong ($R^2 = 0.938$) when the dataset was filtered for Rio Vista flows below 81,101 cfs, the highest flow value that brackets data gaps. Any daily data gaps that could not be filled by the model were imputed with the imputeTS R package (Moritz and Bartz-Beielstein 2017). For the floodplain region, we used dayflow (QYOLO), which models flow from three different input stations in the Yolo Bypass (https://data.cnra.ca.gov/dataset/dayflow). QYOLO is the most accurate source of flow data for the floodplain region because gages are unable to accurately measure flow during inundation.

Shortwave radiation

Daymet (https://daymet.ornl.gov) is a 40-year daily meteorological dataset on a 1 km grid for North America, Hawaii, and Puerto Rico, providing temperature, precipitation, shortwave radiation, vapor pressure, snow water equivalent, and day length based on weather stations included in the National Centers for Environment Information Global Historical Climate Network Daily database (GHCNd). Daymet also includes an objective quantification of uncertainty based on cross-validation analysis for temperature and precipitation (Thornton et al. 2021). We used shortwave radiation data from Daymet extracted for a point in the Yolo Bypass floodplain (latitude: 38.3534, longitude: -121.6543), a point in the mainstem of the Sacramento River (latitude: 38.5319, longitude: -121.528) and a point in the river downstream from the floodplain (latitude: 38.1517, longitude: -121.6883) for the time period of the study. Shortwave radiation is the Incident shortwave radiation flux density in W/m^2, taken as an average over the daylight period. It is commonly used to calculate photosynthetically active radiation (PAR) which is often used as an indicator of chlorophyll-a (Savoy and Harvey 2023).

Water temperature

Goertler and Pien (2022) is a daily water temperature dataset for the Yolo Bypass and Sacramento River at Sherwood Harbor and Rio Vista Bridge. These data were integrated from three sources; water quality data collected during the Yolo Bypass Fish Monitoring Program's (YBFMP) fish collection (IEP et al. 2023), and temperature logger data attached to YBFMP's rotary screw trap and Sherwood Harbor on the Sacramento River (ranged from daily means, in 1998, to a fifteen-minute collection interval, 2013-2017 for the Yolo Bypass, and 2009-2019 for Sherwood Harbor) as well as California Data Exchange Center (CDEC) data at the Lisbon Weir (Pien et al. 2020). Weekly mean of mean daily temperature for each region was included as a main effect in the tested models.

Table S2. Data descriptions for datasets used in the study.

**Covariates hypothesized to influence chlorophyll-a**	**Data metrics**	**Data derivation**	**Locations sampled**	**Time-step**	**Source**
Flow	Discharge	Averaged from 15-minute data; based on river stage and mean velocity or index velocity time-series	Sacramento R. at Verona, Sacramento R. at Rio Vista, Dayflow at Yolo (Yolo Bypass flow at Woodland + Sacramento Weir Spill + South Fork Putah Creek flow)	Daily	NWIS, Dayflow QYOLO
Inundation	1. Inundation (yes/no)<br>2. Inundation duration (number of days of continuous floodplain inundation)<br>3. Inundation factor (none, short, long)	Modeled inundation; based on weir overtopping and flow	Yolo Bypass near Woodland, Fremont Weir, Sacramento Weir, and South Fork Putah Creek	Daily	Inundation R package (Clark and Goertler 2022)
Water temperature	Water temperature	Weekly mean of mean daily water temperature	Sacramento R at Sherwood Harbor, Sacramento R at Rio Vista, Yolo Bypass	Weekly	(Goertler and Pien 2022)
Shortwave Radiation	Incident shortwave radiation flux density in W/m^2, taken as an average over the daylight period	Weekly mean of daily average shortwave radiation	Gridded data for 3 points in the 3 regions, floodplain, mainstem and downstream. See text for co-ordinates	Weekly	(Thornton et al. 2021)

Inundation

We used metrics of floodplain inundation from the “inundation” R package (Clark and Goertler 2022), which calculates the duration of inundation days up to and including that date, among other inundation metrics (Table S2). An inundation event begins when the stage height of the Sacramento River exceeds the height of the Fremont Weir (and thus overflows into the Yolo Bypass) and ends when flow is reduced to within bank of the tidal perennial channel along the Yolo Bypass' eastern edge (e.g., the Toe Drain). We classified values of inundation duration into three categories: none (no inundation), short (less than or equal to 21 days of inundation), and

long (22 days of inundation or more). These cutoffs were created based on a value between the mean and median inundation duration (23.3 days and 17 days, respectively) within the fourteen years in which the floodplain was activated in our study (1999, 2000, 2002, 2003, 2004, 2005, 2006, 2010, 2011, 2013, 2016, 2017, 2018, 2019). Partitioning long and short inundation also yielded the best balance of data points between the two inundation periods (547, 71, 72, for none, short and long, respectively). Figure S1 visualizes inundation periods for all years included in our study and Table S3 shows more detailed information about each event.

Table S3. Inundation summary by water year. Total number of inundation days in one year, the start and end of the inundation period, the maximum duration of any one inundation event occurring in that water year and the number of flooding events (weir overtopping flows). The total and maximum inundation days calculation begins at the weir overtopping and includes the drainage period. No Yolo Bypass inundation occurred in 2001, 2007, 2008, 2009, 2012, 2014 and 2015.

Water Year	Total	Start	End	Max. duration	Number of events
1997	60	12/13/1996	02/18/1997	53	2
1998	100	01/15/1998	06/08/1998	91	3
1999	39	12/06/1998	03/15/1999	35	2
2000	34	02/14/2000	03/18/2000	34	1
2002	15	01/04/2002	01/18/2002	15	1
2003	31	12/31/2002	05/08/2003	29	3
2004	40	01/02/2004	03/14/2004	25	2
2005	6	05/20/2005	05/25/2005	6	1
2006	114	12/24/2005	05/06/2006	67	3
2010	21	01/23/2010	02/12/2010	21	2
2011	35	12/20/2010	04/10/2011	25	3
2013	14	12/04/2012	12/30/2012	7	3
2016	13	03/12/2016	03/24/2016	13	1
2017	112	12/17/2016	05/03/2017	82	4
2018	4	04/08/2018	04/11/2018	4	1
2019	71	01/20/2019	04/20/2019	65	4

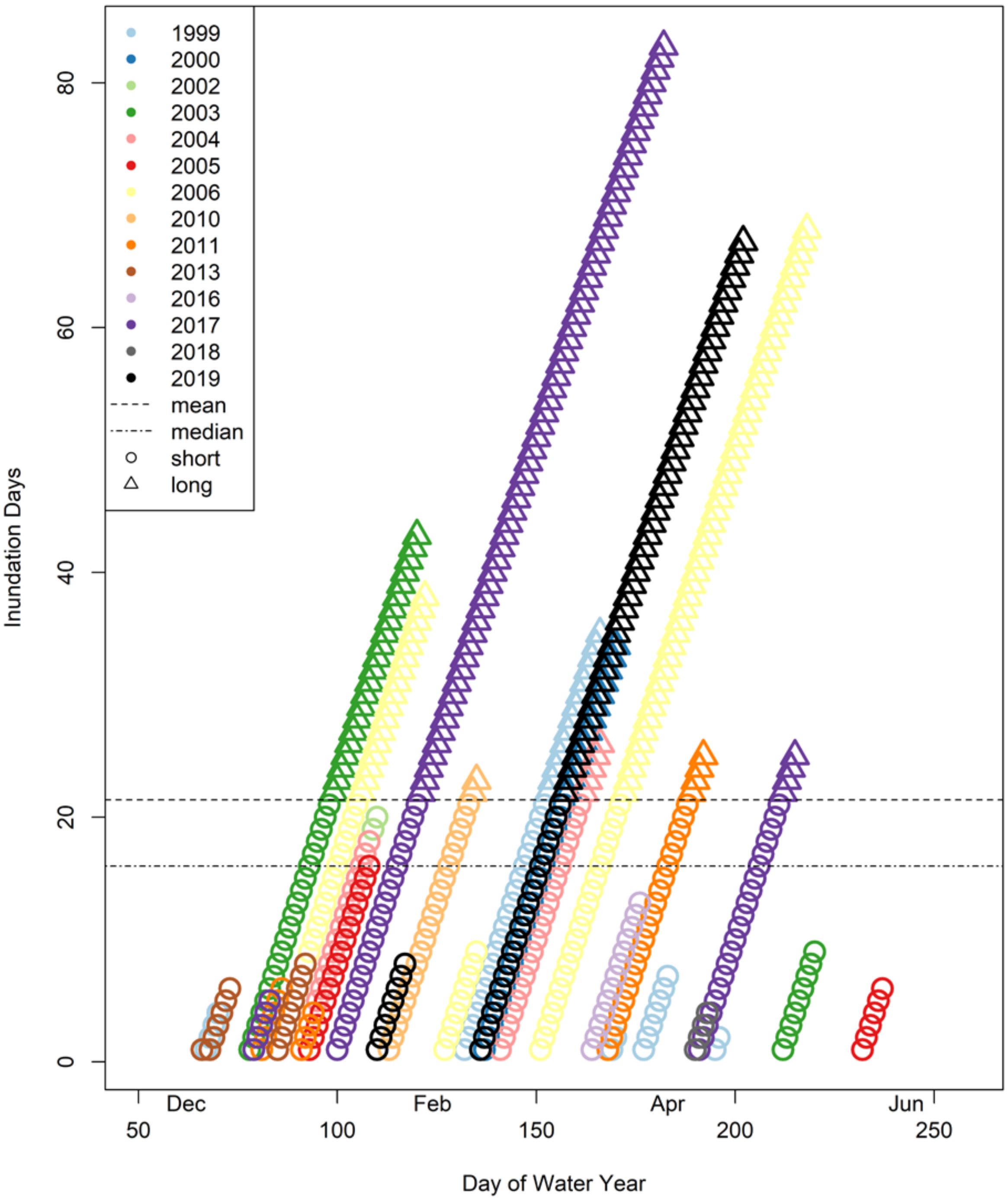

Figure S1. Inundation events for each water year, plotted by day of water year. The mean (21.4 days) and median (16 days) number of days of inundation in the water years included in our study are also shown. Short and long (inundation exceeds 21 days) inundation is denoted by circles and triangles, respectively.

Data Diagnostics and Cleaning

Data diagnostics of the Chlorophyll-a data were assessed following the methods reported in Zuur et al. (2010). The daily Chlorophyll-a data were log-normal distributed, and no outliers were detected, but evidence of temporal autocorrelation was apparent. Homogeneity of variance did not exist across region and the floodplain and downstream regions had higher variance than the mainstem (Figure 2: main manuscript). Data were not zero inflated.

Covariates were also tested for normality and collinearity (Figure S2). Based on the observed distributions of the covariates, only mean daily flow was log-transformed to normalize the distribution (Figure S3). The variance inflation factor was less than three for all covariates included in the final models, and so collinearity was not accounted for.

Daymet simulated shortwave radiation data, and water temperature data collected in the field surveys were collinear ($R^2 > 0.74$; Figure S2). Hence, we tested models based on each of these covariates and found that water temperature produced results with the best fit. Thus, water temperature was included in our final model as one of the covariates and shortwave radiation was excluded.

We explored three different categorizations of floodplain inundation as described in Table S2. Inundation duration as a continuous variable was not feasible to include as a third covariate within the tensor product of the GAM in addition to temperature and flow. All model formulations that included inundation duration did not converge. Hence, we considered two categorical formulations of inundation – one as a simple yes/no flag indicating whether the floodplain was inundated, and the second as a three-category flag indicating “none” (floodplain not inundated), “short”, or “long” duration of flooding (see section on inundation above). Model explorations indicated that the inundation factor with three levels maximized model fit; hence, we selected inundation flag in the final models.

The final covariates that were chosen along with the corresponding value of chlorophyll-a are visualized in Figure S4.

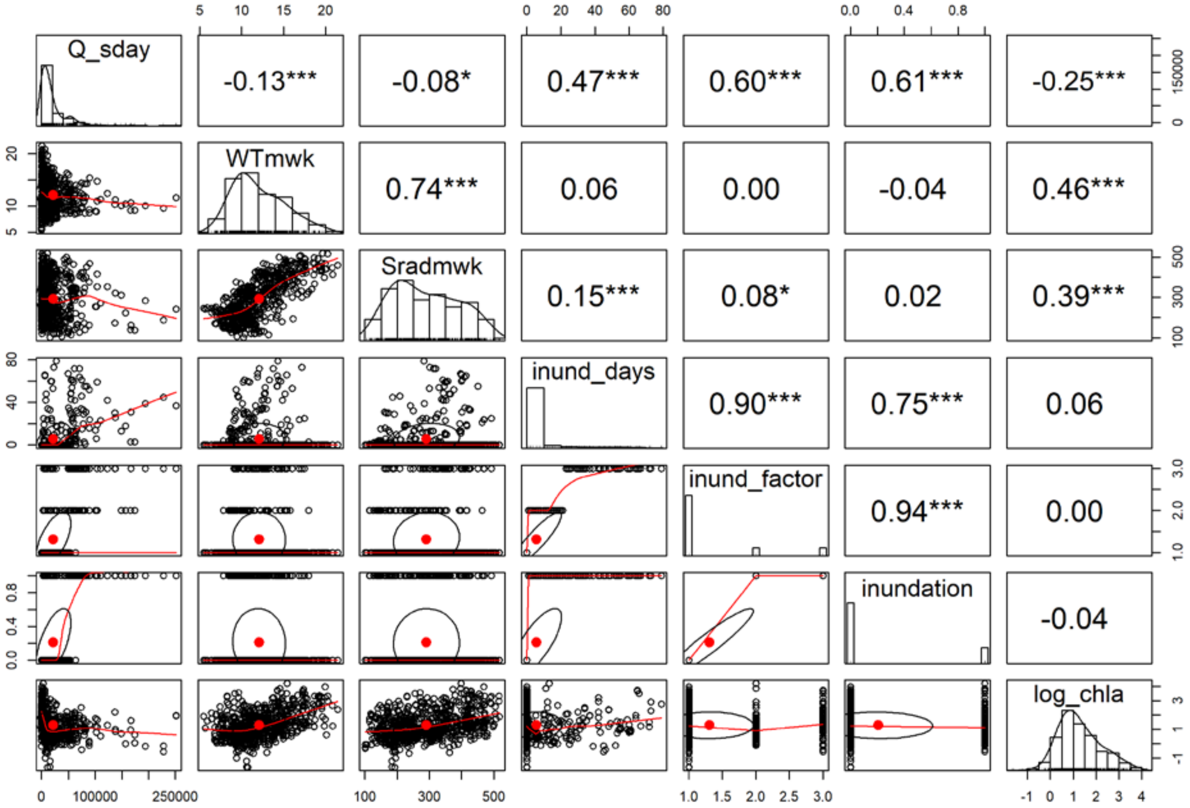

Figure S2. Correlations and Histograms for Covariates and Chlorophyll-a Data. Q_sday: flow on the day of chlorophyll-a measurement; WTmwk: mean weekly water temperature; Sradmwk: mean weekly shortwave radiation; inund_days: duration of floodplain inundation in days; inund_factor: none, short of long inundation period; inundation: binary flag indicating floodplain inundation yes/no; log_chla: log of chlorophyll-a.

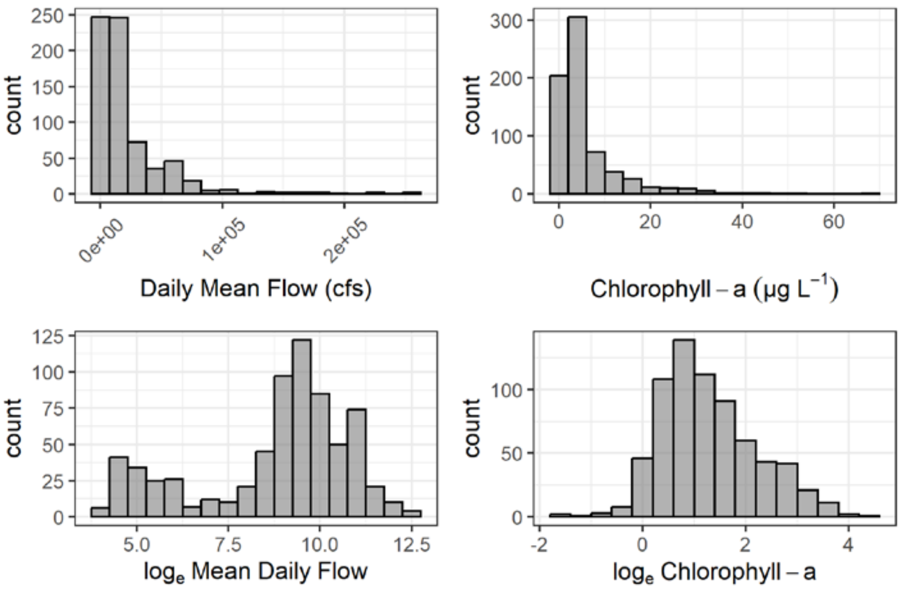

Figure S3. Histograms of transformed and untransformed flow and chlorophyll data.

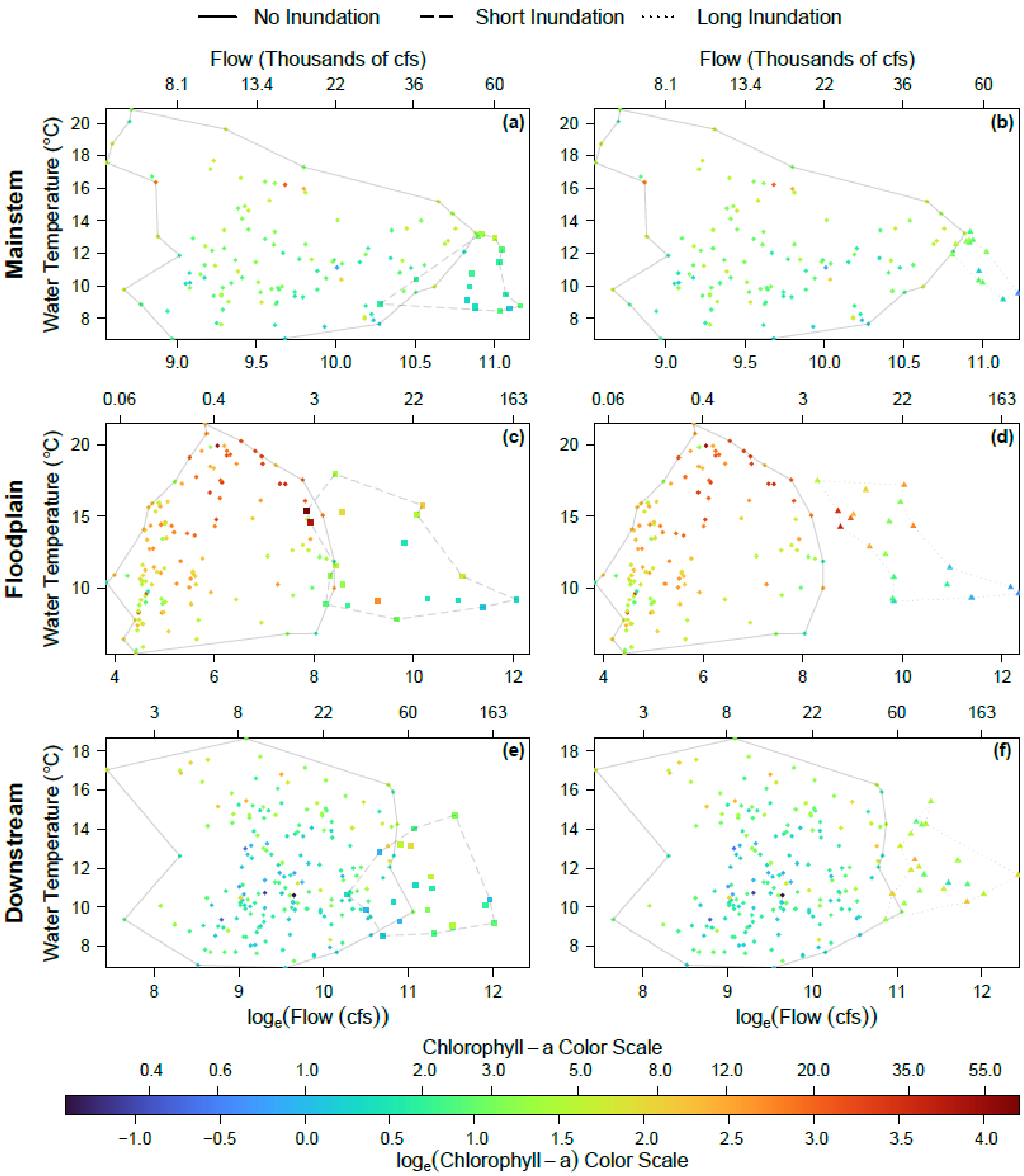

Figure S4. Chlorophyll-a values in µgL-1 for flow and water temperature (a) in **mainstem** for none and short inundation, (b) none and long inundation, (c) in **floodplain** for none and short inundation, (d) none and long inundation, (e) in **downstream** for none and short inundation, (f) and none and long inundation durations. Data points from the input data are plotted as filled circles for no inundation, squares for short inundation and triangles for long inundation periods.

Model validation

Figures S5, S6 and S7 show that model assumptions are being met for the final three models selected for each of the three regions. Table 3 summarizes T values and deviance explained for each of the three regional models.

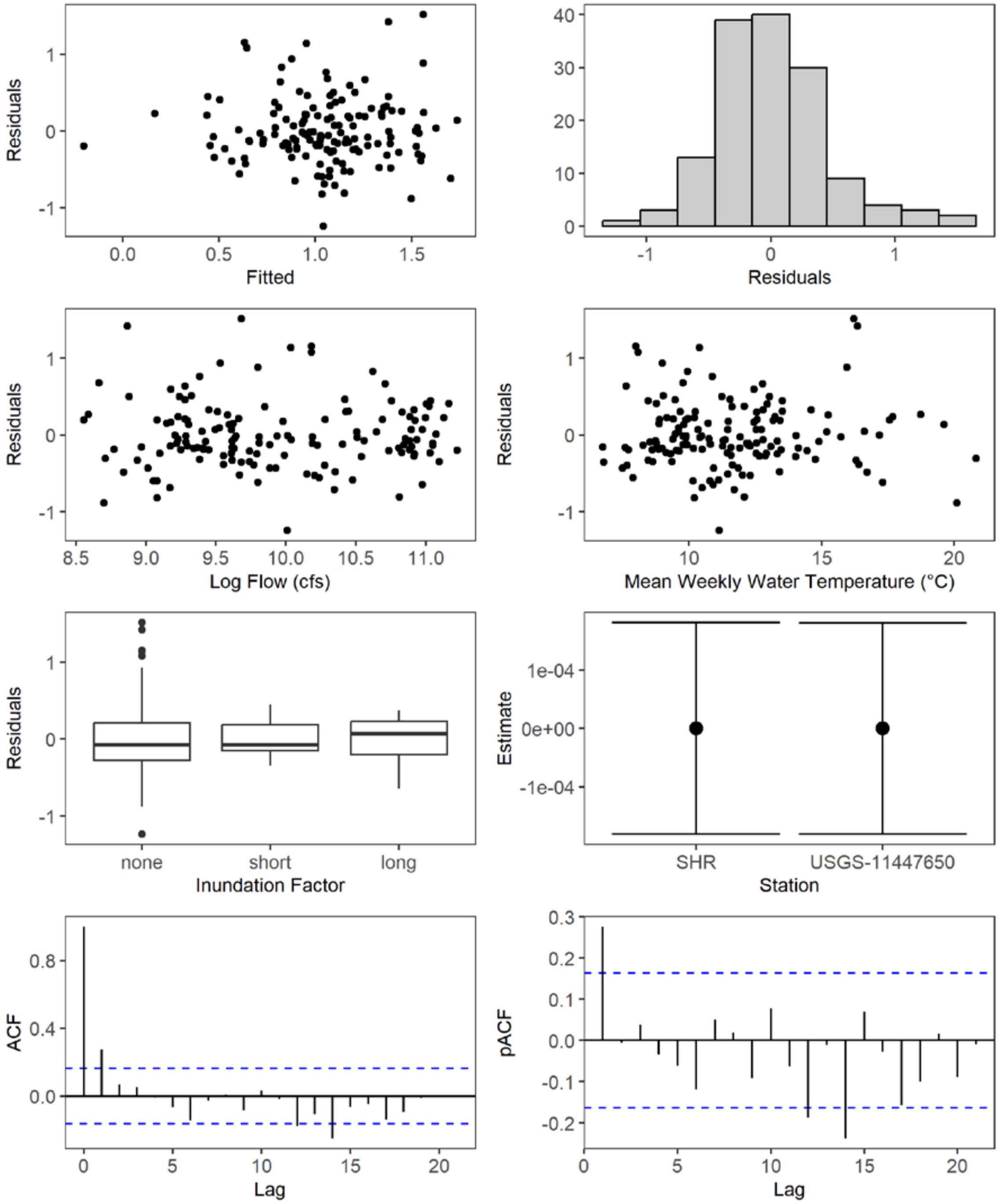

Figure S5. Model Validation Plots for Mainstem Region.

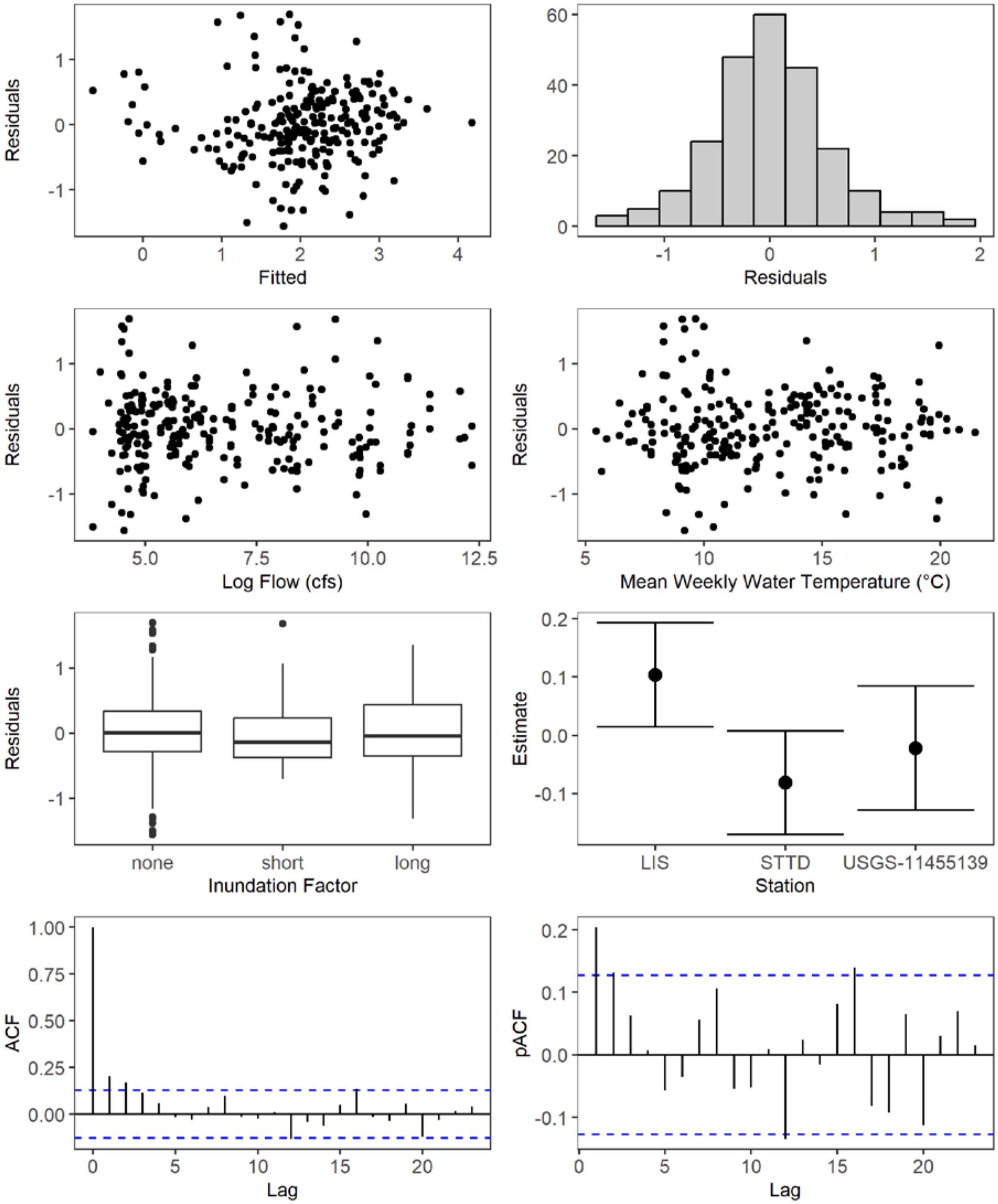

Figure S6. Model Validation Plots for Yolo Floodplain Region.

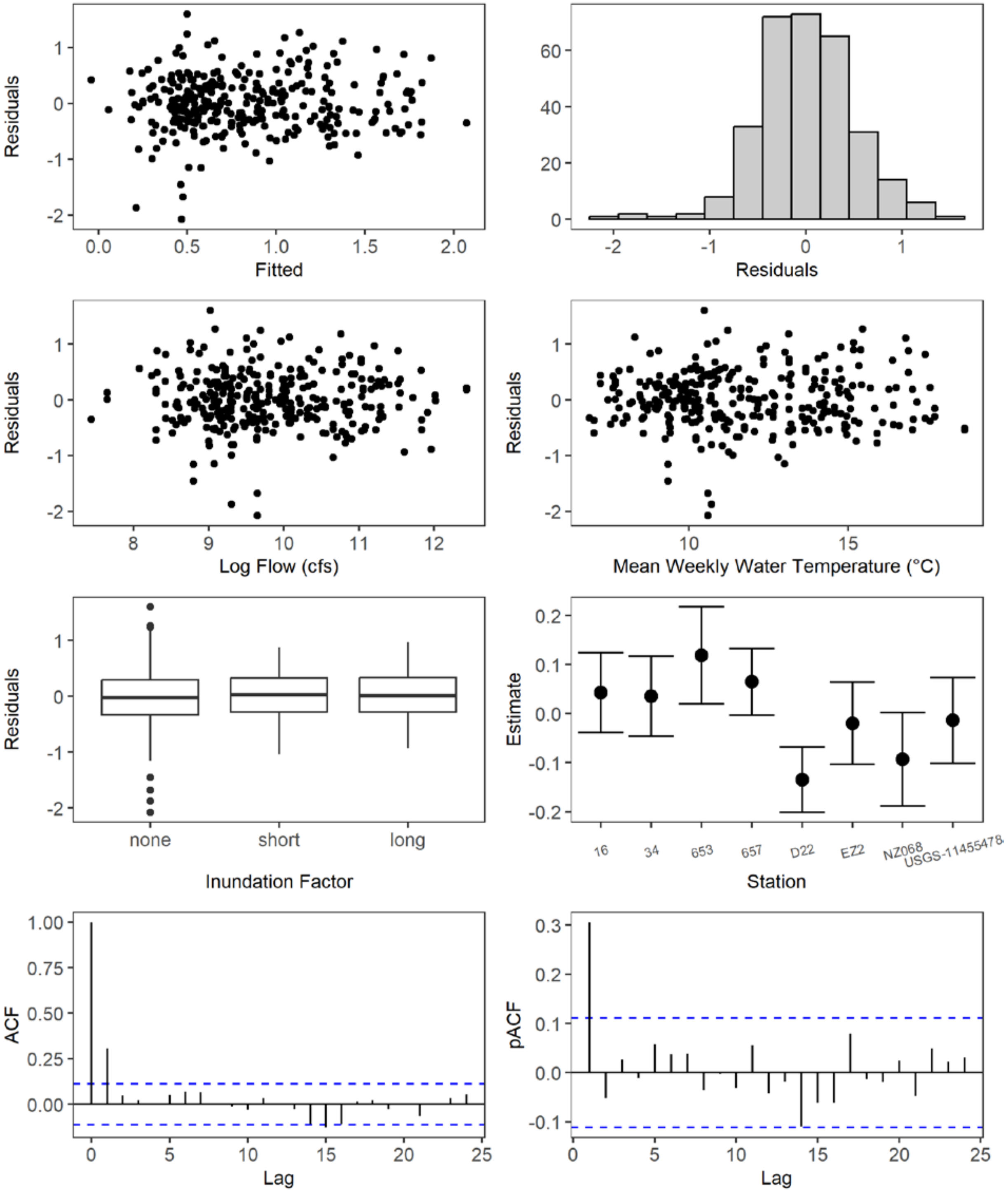

Figure S7. Model Validation for Downstream Region.

References

Bashevkin, S. M., D. Bosworth, S. E. Perry, E. B. Stumpner, and R. Hartman. 2023. decades (1959-2022) of water quality in the upper San Francisco Estuary: an integrated database of 16 discrete monitoring surveys in the Sacramento San Joaquin Delta, Suisun Bay, Suisun Marsh, and San Francisco Bay. ver 7.doi:10.6073/pasta/8dbd29c8c22f3295bbc5d3819fb51d00

De Cicco, L. A., R. M. Hirsch, D. Lorenz, W. D. Watkins, and M. Johnson. 2022. dataRetrieval: R packages for discovering and retrieving water data available from Federal hydrologic web services.doi:10.5066/P9X4L3GE

Clark, J., and P. Goertler. 2022. Inundation.doi:10.5281/ZENODO.6450272

Goertler, P., and C. Pien. 2022. Daily water temperature (C) in the Yolo Bypass and Sacramento River, 1998-2019 ver 2.doi:10.6073/pasta/5d84e5b8ea74dd0854d4aba1e4a6122d

IEP, C. Pien, J. Adams, and N. Kwan. 2023. Interagency Ecological Program: Discrete water quality and phytoplankton data from the Sacramento River floodplain and Yolo Bypass tidal slough, collected by the Yolo Bypass Fish Monitoring Program, 1998 - 2022. ver 1.doi:10.6073/pasta/5791d7eaca09fb9471c5589c66f86863

Moritz, S., and T. Bartz-Beielstein. 2017. imputeTS: Time Series Missing Value Imputation in R. The R Journal **9**: 207–218. doi:10.32614/RJ-2017-009

Pien, C., J. Hamilton, R. Hartman, M. Nelson, J. F. Saraceno, B. M. Schreier, and B. E. Davis. 2020. Hourly water temperature from the San Francisco Estuary, 1986 - 2019, ver 2.doi:10.6073/pasta/7385985f68b02c0deb2a9e425a9f3ad8

Savoy, P., and J. W. Harvey. 2023. Predicting Daily River Chlorophyll Concentrations at a Continental Scale. Water Resources Research **59**. doi:10.1029/2022WR034215

Sommer, T., W. Harrell, M. Nobriga, and R. Kurth. 2001a. Floodplain as Habitat for Native Fish: Lessons from California’s Yolo Bypass. California Riparian Systems: Processes and Floodplain Management, Ecology, and Restoration 81–87.

Sommer, T. R., W. C. Harrell, and M. L. Nobriga. 2005. Habitat Use and Stranding Risk of Juvenile Chinook Salmon on a Seasonal Floodplain. North American Journal of Fisheries Management **25**: 1493–1504. doi:10.1577/m04-208.1

Sommer, T. R., M. L. Nobriga, W. C. Harrell, W. Batham, and W. J. Kimmerer. 2001b. Floodplain rearing of juvenile chinook salmon: evidence of enhanced growth and survival. Canadian Journal of Fisheries and Aquatic Sciences **58**: 325–333. doi:10.1139/cjfas-58-2-325

Thornton, P. E., R. Shrestha, M. Thornton, S. C. Kao, Y. Wei, and B. E. Wilson. 2021. Gridded daily weather data for North America with comprehensive uncertainty quantification. Scientific Data **8**: 1–17. doi:10.1038/s41597-021-00973-0

Zuur, A. F., E. N. Ieno, and C. S. Elphick. 2010. A protocol for data exploration to avoid common statistical problems. Methods in Ecology and Evolution **1**: 3–14. doi:10.1111/j.2041-210X.2009.00001.x
